# 

**Published:** 1858-11

**Authors:** 


### CONTENTS.

ORIGINAL.

Physiological Observations on the Teeth, by Prof. St. John, ........ 105
Letter from J. Lee, M. D., D. D. S. ........ 108
Models by " " " ........ 110
Dental Patents, by J. T. T. ........ 111
Dental News Letter, by Jerome, ........ 113
Setting Pivot Teeth, by J. S. Wingate, ... 115
Savannah, ........ 116
Cincinnati Dental Association by Junius, ........ 120
Inflammation by C. C. Dills, "Correction," 136

SELECTED.

On the Dental movement in England, .... 117
How to secure good teeth, by E. Collins, .. 12
Forbes' Socket Handle, ........ 124
Merry's Drill Stock, ........ 125
CUTTINGS, OBITUARY, NOTICES, &c., ........ 126 to 139

